# Methicillin-resistant Staphylococcus aureus enterocolitis sequentially complicated with septic arthritis: a case report and review of the literature

**DOI:** 10.1186/1756-0500-7-21

**Published:** 2014-01-09

**Authors:** Yukari Ogawa, Takeshi Saraya, Takashi Koide, Ken Kikuchi, Kosuke Ohkuma, Koji Araki, Hiroshi Makino, Shota Yonetani, Hajime Takizawa, Hajime Goto

**Affiliations:** 1 Department of Respiratory Medicine, Kyorin University School of Medicine, 6-20-2 Shinkawa, Mitaka, Tokyo 181-8611, Japan; 2 Department of Infection Control Science, Faculty of Medicine, Juntendo University, Tokyo, Japan; 3 Department of Clinical Laboratory, Kyorin University School of Medicine, Tokyo, Japan

**Keywords:** Methicillin-resistant *Staphylococcus aureus*, Fecal leukocytes, Multilocus sequence typing

## Abstract

**Background:**

Although most reports describing patients infected with methicillin-resistant *Staphylococcus aureus* enterocolitis have been published in Japan, this concept remains a matter of debate and diagnostic criteria have not yet been defined.

**Case presentation:**

The general status of a 74-year-old Japanese man referred to our hospital (day 1) with severe community-acquired pneumococcal pneumonia gradually improved with antibiotic therapy. Thereafter, up to 4 L/day of acute watery diarrhea that started on day 19 was refractory to metronidazole but responded immediately to oral vancomycin. Gram staining stool samples was positive for abundant fecal leukocytes from which dominant methicillin-resistant *Staphylococcus aureus* (10^4^ CFU/mL) were isolated, suggesting methicillin-resistant *Staphylococcus aureus* enterocolitis. High fever with methicillin-resistant *Staphylococcus aureus* bacteremia was evident at day 30, and suppurative right hip arthritis developed around day 71. All methicillin-resistant *Staphylococcus aureus* strains isolated from stools, blood and aspirated synovial fluid separated in the same manner *on p*ulsed-field gel electrophoresis, *as well as two other strains isolated from sputum,* belonged to the same clone as sequence type (ST) 764 (complex clonal 5), and carried SCC*mec* type II.

**Conclusion:**

The clinical, microbiological and molecular biological findings of this patient indicated methicillin-resistant *Staphylococcus aureus* enterocolitis that led to septic methicillin-resistant *Staphylococcus aureus* arthritis.

## Background

We describe a 74-year-old man who was diagnosed with methicillin-resistant *Staphylococcus aureus* (MRSA) enterocolitis that subsequently caused septic arthritis. We reviewed reports of MRSA enterocolitis published in Japan over the past 10 years and characterized the clinical features of MRSA enterocolitis. Here, we discuss the clinical significance of staphylococcal enterotoxins (SEs) and toxic shock syndrome toxin-1 (TSST-1).

## Case presentation

**Figure 1:**
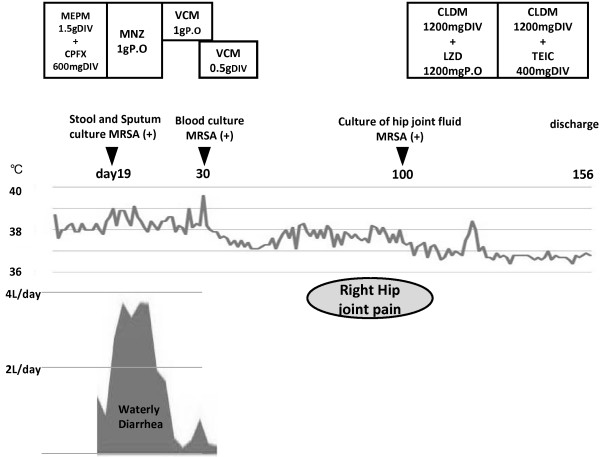
**Clinical course of the patient.** Abbreviations: CLDM, clindamycin; CPFX, ciprofloxacin; DIV, drip infusion; LZD, linezolide; MEPM, meropenem; MRSA, methicillin-resistant *Staphylococcus aureus*; MZN, metronidazole; TEIC, teicoplanin; VCM, vancomycin.

**Figure 2:**
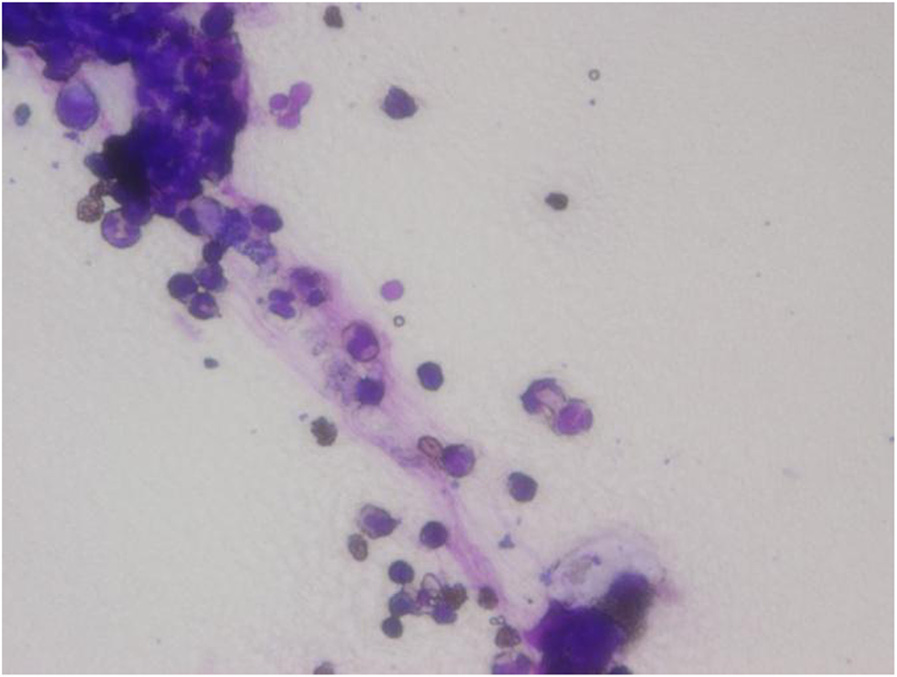
**Gram staining findings of stool cultures.** Fecal leukocytes are abundant.

A 74-year-old Japanese man was transferred to our department from a local hospital under a diagnosis of pneumonia. He had been in good health five days before transfer to our hospital, but with pyrexia and dyspnea upon effort. He had been treated with oral antihyperglycemic drugs to control type 2 diabetes mellitus five years previously. He was a carpenter who did not consume alcohol, but had a smoking history of 44 pack-years. Although he could walk unaided, he seemed very ill. His consciousness level was E4V5M6 (Glasgow coma scale), and his vital signs were: blood pressure, 90/50 mmHg; temperature, 37.3°C; heart rate, 86 beats per min with sinus rhythm; respiratory rate, 24 breaths per minute; and oxygen saturation, 78% with an oxygen supply of 10 L/min delivered via a reservoir mask. He was immediately admitted to the intensive care unit (ICU). Physical findings were normal except for coarse crackles in the bilateral middle to lower lung fields. Upon admission to our hospital (day 1), a chest X-ray revealed infiltration throughout all lung fields except the left upper lung. These results together with positive findings of pneumococcal urinary antigens indicated a diagnosis of pneumococcal pneumonia (CURB 65 score was 3, which is defined as Confusion, Urea nitrogen, Respiratory rate, Blood pressure, and 65 years of age and older; pneumonia severity index was class IV). He was immediately intubated and treated with intravenous meropenem (1.5 g/day) plus ciprofloxacin (600 mg/day), which gradually improved his respiratory status over a period of two weeks. However, acute watery diarrhea amounting to 4 L/day (determined by inserting a rectal tube) together with pyrexia (38°C) was evident on day 19 (Figure 1). Although stool specimens were negative for *Clostridium difficile* toxins A and B, pseudomembranous colitis was considered and metronidazole (1 g/day) was started via a nasogastric tube (Figure 1). However, the watery diarrhea was refractory to metronidazole, and Gram staining stool samples revealed abundant fecal leukocytes (Figure 2), suggesting colonic membranous damage. Although colonic fiberscopy on day 22 provided limited information only as far as the sigmoid portion because the health status of the patient was unstable, definitive evidence of pseudomembranous formation other than membranous edema or hyperemia was not found. Furthermore, methicillin-resistant *Staphylococcus aureus* (MRSA; 10^4^ colony forming unit/mL) was predominantly isolated from stool cultures on day 19 whereas other enteric bacterial pathogens and parasites or ova were negative, indicating MRSA enterocolitis*.* Vancomycin (VCM; 1 g/day) delivered via a nasogastric tube completely resolved the watery diarrhea within a few days. However, at day 30, high fever recurred with moderate watery diarrhea (~500 mL/day), and one of two sets of blood cultures was positive for MRSA. Thereafter, intravenous VCM (0.5 g/day) with dose adjustment for renal dysfunction was initiated, and repeated blood cultures were negative for MRSA. The total amount of watery diarrhea excreted between days 19 and 34 was 22.43 L and the maximum volume per 24 h reached 3.50 L on day 25.

**Figure 3:**
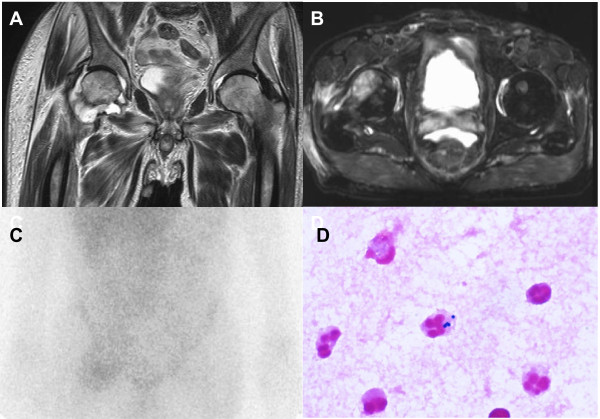
**Magnetic resonance imaging of right hip joint.** T2-weighted image (T2WI) of hip joint shows high-intensity area surrounding head of right femur, indicating fluid collection **(A)**. Fat saturated T2WI shows high-intensity area on right obturator externus or adducent muscles **(B)** that appears as a hotspot on gallium scintigram **(C)**. Synovial fluid contains abundant leukocytes that are phagocytically engulfed by gram-positive cocci **(D)**.

**Figure 4:**
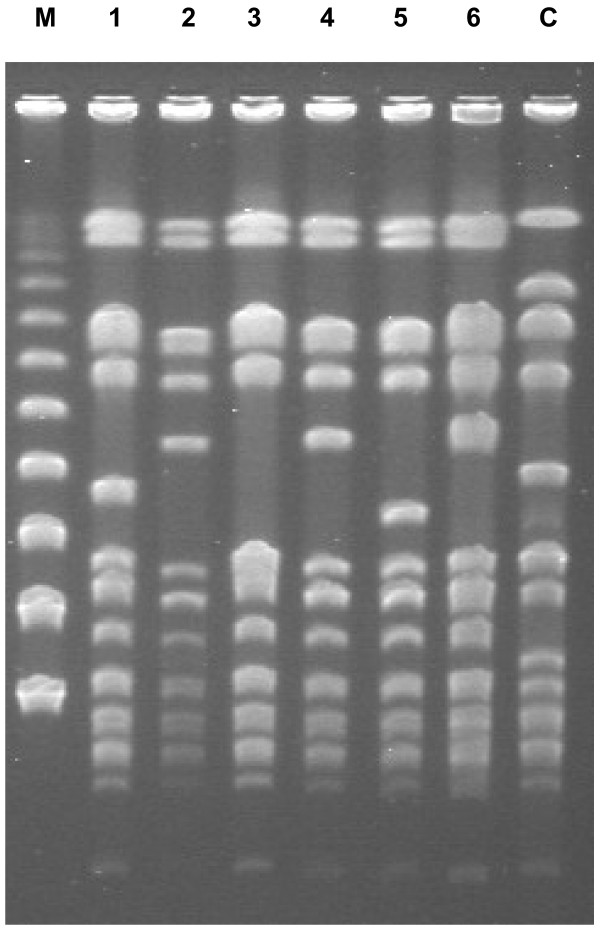
**Analysis of identified methicillin-resistant** ***Staphylococcus aureus*** **strains on pulsed-field gel electrophoresis.** M, marker; 1, sputum (day 10); 2, stool (day 19); 3, intravenous hyperalimentation catheter (day 20); 4, blood (day 30); 5, sputum (day 44); 6, hip joint synovial fluid (day 100); C, control (*Staphylococcus aureus*, strain NCTC 8325).

The general status of the patient gradually improved except for persistent low-grade fever, and he was able to walk unaided by day 64. Intermittent pain in the right hip joint started on day 71 after he started rehabilitation for walking (Figure 1). A physical examination revealed the obturator or psoas sign, and he was positive for the Gaenslen maneuver, suggesting inflammation at the retroperitoneal or lumbar vertebrae and/or sacroiliac joint. We explored the source of the fever and right hip joint pain using magnetic resonance imaging (MRI) (Figure 3A and B) and ^67^gallium scintigraphy of the hip joint. T2-weighted MRI (T2WI) revealed an area of high intensity around the head of the right femur indicating fluid collection (Figure 3A). Fat-saturated T2-weighted images (T2WI) revealed another area of high intensity on the right obturator externus or adducent muscles (Figure 3B), suggesting right hip arthritis that ^67^gallium scintigraphy confirmed as a hotspot (Figure 3C). Joint drainage fluid collected on day 100 showed abundant leukocytes enclosed by phagocytic gram-positive cocci (Figure 3D) that were later identified as MRSA. Pulsed-field gel electrophoresis (PFGE) showed that the MRSA strains in the samples isolated from stool (day 19), blood (day 30) and synovial fluid (day 100) were identical (Figure 4), suggesting a hematological spread of MRSA originating from the colonic membrane damaged by MRSA enterocolitis. The PFGE profiles of two strains isolated from sputum on days 10 and 44 and that of a strain isolated from a catheter tip on day 20 were similar, but all three strains differed by one band from strains isolated from stool, blood and synovial fluid specimens. These strains were considered to be derived from the same clone with different subtypes according to Tenover's criteria [1], and they had the same antibiogram (VCM ≤ 1, teicoplanin ≤ 1, linezolide 2). These findings suggested that persistent colonized MRSA could co-evolve even in a host environment.

None of the findings were consistent with the criteria for confirmed toxic shock syndrome (TSS; fever > 38.9°C, hypotension ≤ 90 mmHg), diffuse erythroderma, desquamation (unless death occurs before desquamation) and the involvement of at least three organ systems) or probable TSS (missing one characteristic for confirmed TSS) [2] or pseudomembranous colitis. Thus, he was finally diagnosed with MRSA enterocolitis that subsequently caused septic arthritis in the right hip.

Indeed, multilocus sequence typing confirmed that the allelic profiles of the MRSA strains obtained from blood, sputum and synovial fluid on days 30, 45 and 100, respectively, were identical (Table 1), belonged to sequence type (ST) 764, clonal complex (CC) 5 [3] and carried SCC*mec* type II. These strains carried enterotoxins G and I, but no other SEs or TSST-1. Our patient was discharged uneventfully on day 156 after completing a two-month course of oral linezolide (1200 mg/day) with intravenous clindamycin (CLDM; 1200 mg/day) followed by teicoplanin (400 mg/day) with intravenous CLDM (1200 mg/day) infusion.

**Table 1:** Toxin gene profiles and multilocus sequence typing of MRSA strains obtained from blood, sputum, and synovial fluid

**Source**	**Detection of toxic genes**													**SCC** ***mec***			**MLST**								
	***tst***	***eta***	***etb***	***sea***	***seb***	***sec***	***sed***	***see***	***seg***	***seh***	***sei***	***sej***	***pvl***	***mec*** **type**	***ccr*** **type**	**SCC** ***mec***	***arcC***	***aroE***	***glpF***	***gmk***	***pta***	***tpi***	***yqil***	**ST**	***CC***
A. blood	-	-	-	-	-	-	-	-	+	-	+	-	-	A	2	II	1	136	1	4	12	1	10	764	5
Sputum	-	-	-	-	-	-	-	-	+	-	+	-	-	A	2	II	1	136	1	4	12	1	10	764	5
Joint fluid	-	-	-	-	-	-	-	-	+	-	+	-	-	A	2	II	1	136	1	4	12	1	10	764	5

A: acetyltransferase, *arc*: carbamate kinase, *aro*: shikimate dehydrogenase, CC: clonal complex, *ccr*: cassette chromosome recombinase, *eta*: exfoliatin toxin a *etb*: exfoliatin toxin b, *glp*: glycerol kinase, *gmk*: guanylate kinase, MLST: multilocus sequence typing, *pta*: phosphate acetyltransferase, *pvl*: Panton-Valentine leukocidine, *sea*: staphylococcal enterotoxin a, *seb*: staphylococcal enterotoxin b, *sec*: staphylococcal enterotoxin c, *sed*: staphylococcal enterotoxin d, *seg*: staphylococcal enterotoxin g, *seh*: staphylococcal enterotoxin h, *sei*: staphylococcal enterotoxin I, *sej*: staphylococcal enterotoxin j, SCC*mec*: Staphylococcal casstte chromosmome *mec,* ST: sequence type, *tpi*: triosephosphate isomerase, *yqi*: acetyle coenzyme.

## Discussion

MRSA enterocolitis has been identified among Japanese patients since 1985, especially after undergoing gastric resection [4] or being medicated with a third-generation cephalosporin [5]. Although many studies of MRSA enterocolitis have proceeded in Japan, MRSA is not a frequent cause of antibiotic-associated diarrhea and this notion has remained a matter of debate, as anecdotally reported elsewhere [6]. Therefore, to summarize and characterize published reports of MRSA enterocolitis in Japan, we intensively reviewed studies over the past decade (Table 2) [7-24]. We identified 36 (male, n = 28; female, n = 8) patients (age range, 0 to 91 y; mean ± SD; 59.5 ± 21.3 y) who required a median of seven (range, 2–40) days of antibiotics to treat MRSA enterocolitis that was mainly diagnosed from stool cultures. Two were diagnosed at autopsy (patients 1 and 3) and one each was diagnosed by colonoscopy (patient 2) and during surgery (patient 4). Importantly, SEs or TSST-1 have been assessed in only four (11.1%) patients (Nos. 1, 5, 6 and 8); two of them had enterotoxin C and all four had TSST-1. Furthermore, our review of two reports [12,13] found that patients No. 5 and 6 (Table 2) satisfied the criteria for TSS with typical erythema and desquamation, but the authors had diagnosed them with MRSA enterocolitis. The other two patients (Nos.1 and 8) with TSST-1 did not satisfy the criteria for TSS. None of the other patients with MRSA enterocolitis had cutaneous lesions similar to those of TSS. MRSA colitis clinically resembles TSS in many respects, such as shock, multiple organ failure and pyrexia due to MRSA bacteremia, but it should be differentiated from TSS regardless of the presence of TSST-1.

**Table 2:** **Summary of methicillin-resistant** ***Staphylococcus aureus*** **enterocolitis reported in Japan over the past 10 years**

**Pt. No.**	**Age**	**Sex**	**Underlying disease**	**Diagnostic method**	**Site of infection**	Duration* (days)	**Enterotoxin/coagulase Type/TSST-1**	**Prog.**	**Ref**
1	66	M	Appendectomy	Autopsy	All digestive tract	NA	NA/II/+	D	9
2	31	M	Acute pancreatitis	Stool culture with Biopsy	Colon	12	NA	D	10
3	47	F	UTI	Autopsy	Small Intestine	10	NA	D	7
4	73	M	Esophageal Ca, Gastric CA POS	Ileum interposition	none	19	NA	A	11
5	59	M	Gastric Ca	Stool culture	NA	NA	C/NA/+	A	12
6	23	M	Infected pilonidal sinus POS	NA	NA	15	-/-/+	A	13
7	71	M	Peritonitis with mediastinitis POS	NA	NA	18	NA	A	14
8	72	M	Panperitonitis POS	Stool culture	NA	3	A/II/+	A	15
9	26	F	Tongue Ca POS	Stool culture	NA	7	NA	D	16
10	75	F	Gallbladder Ca	NA	NA	NA	NA	D	8
11	64	M	Pancreatitis	Stool culture	NA	NA	NA	A	17
12	73	F	Pancreatitis	Stool culture	NA	NA	NA	A	17
13	44	M	Pancreatitis	Stool culture	NA	NA	NA	D	17
14	77	M	Pneumonia	Stool culture	NA	42	NA	A	18
15	78	M	Ileus POS	Stool culture	NA	20	NA	A	19
16	78	F	Intestinal perforation POS	Stool culture	NA	NA	NA	D	20
17	39	M	Acute mandibular periostitis	Stool culture	NA	8	NA	A	21
18	60	M	Lung Ca small intestine metastasis POS	NA	NA	7	NA	A	22
19	84	M	Pneumatosis cystoides intestinalis	Stool culture	NA	NA	NA	A	23
20	71	M	AMI, CABG	Stool culture	NA	3	NA	A	24
21	73	M	Sigmoid colon Ca POS	Stool culture	NA	2	NA	A	
22	63	M	Rectum Ca POS	Stool culture	NA	2	NA	A	
23	78	M	UTI	Stool culture	NA	4	NA	A	
24	91	F	Pneumonia, cholecystitis	Stool culture	NA	NA	NA	A	
25	59	M	Esophageal Ca POS	Fecal properties	NA	4	NA	A	
26	70	F	Perforation of sigmoid colon Ca POS	Stool culture	NA	4	NA	A	
27	64	M	Perforation of amebic colitis POS	NA	NA	4	NA	A	
28	0	M	BA	Stool culture	NA	7	NA	A	
29	18	M	Lymphoblastic lymphoma T-cell type	Stool culture	NA	NA	NA	A	
30	64	M	Renal abscess	NA	NA	NA	NA	A	
31	88	F	Pneumonia	NA	NA	9	NA	A	
32	64	M	Pancreatic Ca POS	Stool culture	NA	NA	NA	A	
33	62	M	Ileus	Fluid drainage from ileus tube	NA	5	NA	A	
34	66	M	Esophageal Ca POS	NA	NA	NA	NA	A	
35	26	M	ALL	Stool culture	NA	14	NA	D	
36	47	M	Ileus	NA	NA	9	NA	D	

A, alive; ALL, acute lymphocytic leukemia; AMI, acute myocardial infarction; BA, bronchial asthma; Ca, carcinoma; CABG, coronary artery bypass grafting; D, dead; Duration*, duration of preceding antibiotics therapy to diagnosis of MRSA enterocolitis; NA, not available; POS, postoperative status; Prog, prognosis; Pt. No, patient number; TSST-1, toxic shock syndrome toxin-1; UTI, urinary tract infection; All patients were described in Japanese and only patients 1 to 20 have been published in English and are cited as references.

Although enterotoxins (A, A and B, D [6] or C [25,26], and the bicomponent leukotoxin LukE-LukD [27]) have been detected in strains associated with MRSA enterocolitis, the relevance to this disease of enterotoxins G and/or I that were recognized in our patient remains unknown. Humans are natural reservoirs of *S. aureus*, with intermittent colonization occurring in 30% - 50% of healthy adults [28], 10% of whom harbor this microorganism in the gastrointestinal tract [29]. The ratio of nasal MRSA carriage among healthy individuals in Japan is similarly high, at 38.0% [30]. In contrast, another report stated that most hospitalized geriatric patients carrying MRSA in the gastrointestinal space were treated with antibiotics [31]. These facts imply that most of the reported "MRSA enterocolitis" in Japan might have been caused by preceding antibiotic use and/or presented with one of the clinical features of superantigen-derived toxic shock syndrome due to TSST-1 or undetermined effects of SEs other than those described above. In addition, colonization of MRSA in the digestive tract after antibiotic use might have been misdiagnosed in some patients as a pathogen for diarrhea of undetermined causes.

Boyce *et al*. [6] reported that among five patients with MRSA enterocolitis in whom fecal leukocytes were examined, two were positive, like our patient. They also stated that the duration of preceding antimicrobial therapy ranged from 2 to 21 days, which is similar to that of other Japanese reports indicating a range of 2 to 42 days (Table 2). Boyce *et al*. [6] also reported that the maximum recorded stool volume per 24 h period ranged from 475 to 8,250 mL in 11 patients with MRSA enterocolitis and that the total volume of stool recorded for the entire diarrheal illness ranged from 745 mL to 38.85 L, which was identical to that of our patient and other reports (maximum recorded stool volume per 24 h ranged from 500 mL to 4.0 L from patient Nos. 9, 15, 33 and the present patient). Froberg *et al*. [32] reported that MRSA enterocolitis features loosely adherent pseudomembranes in the small intestine, whereas *C. difficile* pseudomembranous colitis locates in the colon where pseudomembranes are tightly adherent. Indeed, the sites of inflammation in patients 1, 2, 3 and 4 in our review comprised the gastric wall (patient 1 at autopsy), small intestine (patient 3 at autopsy; patient 4 by biopsy), or colon (patient 2, by biopsy), indicating that the preferred site of MRSA enterocolitis is the small intestine. Cultured samples from all four patients were positive for MRSA, but only patients 3 and 4 had white pseudomembranes at the small intestine that were MRSA-positive. Although pathological information is scant, MRSA enterocolitis can involve not only the small intestine, but also the colon (Table 2).

Colonic fiberscopy indicated that our patient had no apparent pseudomembranes up to the sigmoid portion. Nonetheless, MRSA enterocolitis was the most likely source of the massive diarrhea because *C. difficile* toxin A/B was negative, MRSA was the dominant organism isolated from abundant fecal leukocytes, the watery diarrhea was refractory to metronidazole but susceptible to oral VCM and suppurative MRSA arthritis of the right hip joint occurred after the watery diarrhea or transient MRSA bacteremia were identified. Watanabe *et al*. [33] found identical (69.2%) or similar (23.1%) PFGE profiles in 12 of 13 samples obtained from the respiratory tract (nasal cavity and/or pharynx and/or sputum) and stool samples from patients infected with MRSA. They speculated that one cause of enterocolitis could be MRSA that originated from colonization of the respiratory tract and was absorbed into the digestive tract. A single genetic event in MRSA strains colonizing the respiratory tract might generate a new MRSA strain that could cause enterocolitis, septicemia and septic arthritis. As the toxin gene profiles among strains from blood, synovial fluid and sputum were identical on MLST (SCC*mec* type II, ST764, CC5), whether or not a single minor genetic change could alter virulence remains uncertain. Further studies are required to clarify the reason for the change in virulence.

The MRSA strain derived from stool cultures did not thrive. Thus, we could not perform MLST after obtaining convincing PFGE results. Nevertheless, we postulate that our patient had MRSA enterocolitis that was sequentially complicated with septic arthritis. The relationship between MRSA enterocolitis and SEs or TSST-1 together with pathological assessments that could improve understanding of the pathological process involved in MRSA enterocolitis require further exploration.

## Conclusion

Any MRSA isolated from patients with diarrhea that is refractory to treatment should be assessed from a multidisciplinary viewpoint considering colonization or pathogens. Our review of published reports suggests that the clinical features MRSA enterocolitis mimic those of TSS, which might reflect undetermined effects of SEs or TSST-1 associated with MRSA infection.

## Consent

Written informed consent was obtained from the patient for publication of this Case Report and any accompanying images. A copy of the written consent is available for review by the Editor-in-Chief of this journal.

## Abbreviations

CC: Clonal complex; CLDM: Clindamycin; Luk: Leukocidin; MLST: Multilocus sequence typing; MRI: Magnetic resonance imaging; MRSA: Methicillin-resistant *Staphylococcus aureus*; PFGE: Pulsed-field gel electrophoresis; SEs: Staphylococcal enterotoxins; ST: Sequence typing; TSST-1: Toxic shock syndrome toxin-1; VCM: Vancomycin.

## Competing interests

The authors declare that they have no competing interests.

## Authors’ contributions

YO, TS, TK, and KO mainly managed the patient. YO, TS, HT, and HG wrote the manuscript. KK, KA, SY, and HM performed microbiological analyses. All authors read and approved the final manuscript.
